# Impact of transferrin saturation on cardiovascular events in non-dialysis-dependent chronic kidney disease patients treated with darbepoetin alfa

**DOI:** 10.1007/s40620-024-02000-y

**Published:** 2024-06-28

**Authors:** Kentaro Nakai, Tomoya Nishino, Tatsuo Kagimura, Ichiei Narita

**Affiliations:** 1 Division of Nephrology and Dialysis Center, Japanese Red Cross Fukuoka Hospital, 3-1-1 Ogusu, Minami-ku, Fukuoka, 815-8555 Japan; 2 Department of Nephrology, Nagasaki University Hospital, Nagasaki, Japan; 3 Translational Research Center for Medical Innovation, Kobe, Japan; 4 Division of Clinical Nephrology and Rheumatology, Niigata University Graduate School of Medical and Dental Sciences, Niigata, Japan

**Keywords:** Anemia, Cardiovascular disease, Chronic kidney disease, Iron, Transferrin saturation

## Abstract

**Background:**

Although the widespread use of long-acting erythropoiesis-stimulating agents (ESAs) has facilitated the improvement of anemia in patients with chronic kidney disease (CKD), the improvement in prognosis has not been fully demonstrated. Iron deficiency is associated with the development of cardiovascular diseases (CVDs), and the relative iron deficiency induced by erythropoiesis-stimulating agents may prevent the improvement of prognosis. Therefore, we investigated the association between iron deficiency and cardiovascular events during long-acting erythropoiesis-stimulating agent therapy using transferrin saturation (TSAT), which is less susceptible to inflammation than ferritin.

**Methods:**

This study included 1040 patients with non-dialysis-dependent CKD, aged ≥ 20 years, with a glomerular filtration rate < 60 mL/min/1.73 m^2^ and hemoglobin < 11 g/dL, who were treated with darbepoetin alfa for 96 weeks. The patients were recruited in the BRIGHTEN Trial, a multicenter, prospective, observational study conducted to evaluate erythropoiesis-stimulating agent resistance to darbepoetin alfa in treating anemia in non-dialysis-dependent CKD in a clinical setting. The association between transferrin saturation and the cumulative incidence of cardiovascular events was evaluated using the Kaplan–Meier method. To calculate the hazard ratio (HR), 95% confidence intervals (CI) and the Cox proportional hazards model were used.

**Results:**

Survival curve analysis for cardiovascular events indicated that patients with transferrin saturation ≥ 30% had a significantly better prognosis, with an adjusted hazard ratio of 0.34 (95% confidence interval 0.22–0.52). Stratified analysis revealed that patients with transferrin saturation of 30–40% had a significantly lower risk of cardiovascular events than those with transferrin saturation of 20–30%, even after a multivariate-adjusted hazard ratio of 0.33 (95% confidence interval 0.21–0.54).

**Conclusion:**

Patients with CKD and transferrin saturation of 30–40% had significantly fewer cardiovascular events than those with transferrin saturation of 20–30% among patients treated with long-acting erythropoiesis-stimulating agents. Therefore, it may be useful to maintain higher transferrin saturation from the viewpoint of erythropoiesis-stimulating agent responsiveness and the reduction of cardiovascular events.

**Graphical Abstract:**

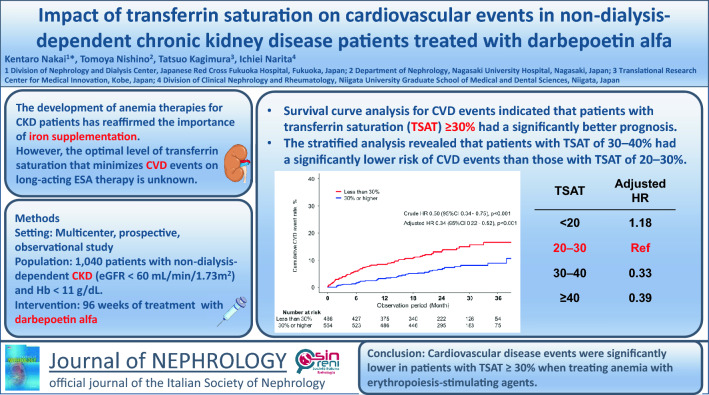

**Supplementary Information:**

The online version contains supplementary material available at 10.1007/s40620-024-02000-y.

## Introduction

Anemia has been reportedly associated with renal prognosis and life expectancy in chronic kidney disease (CKD) patients [1, 2]. Decreased renal function leads to anemia and consequently to a vicious cycle of worsening renal and life prognoses. Although erythropoiesis-stimulating agents (ESAs) are currently widely used to treat anemia in patients with CKD and can even normalize hemoglobin (Hb) without relying on blood transfusions, there are concerns that therapeutic interventions may be paradoxically associated with cardiovascular disease (CVD) as erythropoiesis-stimulating agent doses increase [3]. In an observational study of the 2-year prognosis using the national data of 194,698 dialysis patients, the use of long-acting erythropoiesis-stimulating agents at relatively high doses (24.9 μg/week darbepoetin and 98.1 μg/month epoetin β-pegol) was associated with a significantly worse life expectancy compared to conventional erythropoietin [4]. Erythropoiesis-stimulating agent hyporesponsiveness, which requires a higher dose of erythropoiesis-stimulating agent to reach the target hemoglobin, is an independent, poor prognostic factor [5], and iron deficiency has been reported to contribute to erythropoiesis-stimulating agent hyporesponsiveness [6]. In patients undergoing dialysis, ferritin and transferrin saturation (TSAT) have been reported to be associated with erythropoiesis-stimulating agent responsiveness [7]; iron supplementation has been shown to be an independent factor that results in favorable erythropoiesis-stimulating agent responsiveness in patients with CKD [8]. Adequate iron supplementation may prevent inappropriate increases in erythropoiesis-stimulating agent doses, and Kidney Disease: Improving Global Outcomes (KDIGO) recommends iron supplementation for anemia in patients with CKD with transferrin saturation ≤ 30% and ferritin ≤ 500 ng/mL [9], and the Japanese Society for Dialysis Therapy (JSDT) has suggested that iron supplementation should be preceded by ferritin < 50 ng/mL in patients not receiving erythropoiesis-stimulating agents and ferritin < 100 ng/mL and transferrin saturation < 20% in those receiving erythropoiesis-stimulating agents [10].

Iron overload is an issue when anemia in patients with CKD, including those undergoing dialysis, is treated with blood transfusions. With the widespread use of long-acting erythropoiesis-stimulating agents and the use of hypoxia-inducible factor-prolyl hydroxylase (HIF-PH) enzyme inhibitors, iron has become relatively insufficient. In addition to oral iron supplements, iron-containing phosphorus adsorbents are currently used in patients with CKD, increasing the need for iron sufficiency and prognosis studies. Baseline data of our study indicated that in patients with non-dialysis-dependent CKD, ferritin levels > 500 ng/mL were rarely present, and more than half of the patients had levels < 100 ng/mL [8]. Moreover, > 20% of patients have transferrin saturation < 20%, approximately 70% have transferrin saturation < 30%, and iron supplementation is infrequent and mostly administered orally. Therefore, based on data from the oBservational clinical Research In chronic kidney disease patients with renal anemia: renal proGnosis in patients with Hyporesponsive anemia To Erythropoiesis-stimulating agents, darbepoetiN alfa (BRIGHTEN trial), we aimed to determine the target transferrin saturation levels to reduce cardiovascular events in patients with CKD treated for anemia with long-acting erythropoiesis-stimulating agents.

## Materials and methods

### Study design

The BRIGHTEN Trial is a multicenter, prospective, observational study conducted to evaluate erythropoiesis-stimulating agent resistance to darbepoetin alfa in treating anemia in non-dialysis-dependent CKD in a clinical setting [11]. The study was registered at ClinicalTrials.gov (NCT02136563) and UMIN-CTR (UMIN000013464). Briefly, darbepoetin alfa was administered at 30 μg within 8 weeks of enrollment, and if anemia showed a tendency to improve every 2 weeks, it was subsequently administered once every 4 weeks. Darbepoetin alfa and iron supplementation were adjusted to maintain Hb > 11 g/dL according to the guidelines, and the patients were observed for at least 96 weeks from the start of darbepoetin alfa treatment. The study protocol was approved by the ethics committee of the main institution (Nagoya University, no. 2014-0027) and of each institution (Niigata University, no. 2023-0014; Japanese Red Cross Fukuoka Hospital, no. 23021), and written informed consent was obtained from all participants. This study was conducted in accordance with the 1964 Declaration of Helsinki and its amendments and the Ministry of Health, Labor, and Welfare’s Ethical Guidelines for Clinical Research and under the Japanese Health Insurance System.

### Study population

Of the 1980 patients who were enrolled in 168 facilities, 256 were excluded mainly because of insufficient data on Hb values at 0 and 12 weeks. As shown in Supplemental Fig. 1, this study evaluated 1724 patients and compared the prognosis of 1040 patients between the two groups categorized according to the average value of transferrin saturation levels over the entire observational period (670 patients were excluded because their transferrin saturation was never measured, and 14 patients were excluded because they had no transferrin saturation evaluation before the cardiovascular event). As detailed elsewhere, the inclusion criteria were as follows: age ≥ 20 years with an estimated glomerular filtration rate (eGFR) < 60 mL/min/1.73 m^2^ and Hb < 11.0 g/dL at the most recent examination within 8 weeks prior to enrollment and who had started treatment with darbepoetin alfa [11].

### Measurements

Since this was an observational study, the temporal relationship between the timing of iron administration and the transferrin saturation and ferritin measurements was not specified, and transferrin saturation and ferritin were measured as part of the blood test measurements at the time the subjects came to the hospital. Erythropoietin resistance index (ERI) was calculated using the following equation: ERI = dose of darbepoetin alfa at 12 weeks (μg)/concentration of hemoglobin (g/dL) at 12 weeks.

### Outcomes

The endpoint of this study was defined as the occurrence of cardiovascular events. A fatal cardiovascular event was defined as death due to myocardial infarction, congestive heart failure, arrhythmia, cerebrovascular disease, aortic dissection, other forms of cardiovascular disease, ischemia in major organs, and sudden death. A nonfatal cardiovascular event was defined as hospitalization due to myocardial infarction, angina pectoris, ischemic heart disease requiring invasive treatment, congestive heart failure, severe arrhythmia, atrial fibrillation, atrial flutter, aortic dissection, and ischemia of major organs.

### Statistical analysis

Continuous and categorical variables are shown as mean ± standard deviation and as frequencies and percentages (%), respectively. To compare the distribution of baseline characteristics between the two groups, constituted by the mean value of transferrin saturation during the observation period, the Wilcoxon rank-sum test for continuous variables and the *χ*^2^ test for categorical variables were used. The receiver operating characteristic (ROC) curves for the occurrence of the events were created using the average value of observed values for ferritin and transferrin saturation from baseline to 63 weeks or until event occurrence, and optimum cut-off values of ferritin and transferrin saturation were obtained from the ROC curves. Cumulative event rates for cardiovascular and renal events were calculated using the Kaplan–Meier method. To calculate the hazard ratio (HR), 95% confidence intervals (CI) and the Cox proportional hazards model were used. Adjusted HR was calculated using a multiple Cox proportional hazards model with the following covariates: age, sex, diabetes, body mass index (BMI), etiology of CKD, smoking status, coronary artery disease, heart failure, stroke, peripheral artery disease, renin–angiotensin–aldosterone (RAS) inhibitors, iron supplementation, and laboratory data at baseline (eGFR, Hb, albumin, high-sensitivity C-reactive protein (CRP), N-terminal-proB-type natriuretic peptide (NT-proBNP), glycated hemoglobin (HbA1c), and urinary protein to creatinine ratio). Statistical significance was defined as *P* = 0.05, with a two-tailed test, and the multiplicity of tests was not considered. All data were statistically analyzed using SAS version 9.4 (SAS Institute, Inc., Cary, NC, USA).

## Results

The baseline data of the patients has been previously reported in detail [8]. Mean age was 69.9 years, and 58.8% of the population was male, while average BMI was 23.2. Diabetic kidney disease, nephrosclerosis, and nephritis accounted for 27.7%, 23.5%, and 23.2%, respectively. Iron was administered to 14.5% of the patients at study initiation. The percentage of prescriptions peaked at 12 weeks and then decreased; ferrous citrate accounted for most of the prescriptions (shown in Supplemental Fig. 2). In patients with low ferritin levels at baseline, iron was prescribed, and ferritin levels increased after 12 weeks, reached a plateau after 24 weeks, and followed a course similar to that of patients without iron supplementation (shown in Supplemental Fig. 3a). However, transferrin saturation levels gradually increased until approximately 12 weeks, rapidly increased from 12 to 24 weeks, and reached a plateau after 24 weeks, which occurred similarly to that of ferritin. However, it differed from ferritin because it stabilized at a level higher than the baseline (approximately 35%). In patients with low transferrin saturation levels at baseline, iron was prescribed, and their transferrin saturation levels increased after 12 weeks and reached a plateau after 24 weeks, which is similar to that in subjects without iron supplementation (shown in Supplemental Fig. 3b). After excluding 684 patients with insufficient transferrin saturation data, the remaining 1040 patients experienced 103 events, including 35 hospitalizations for congestive heart failure, 18 for cerebrovascular disease, 12 for angina pectoris, and 8 for ischemic heart disease requiring invasive intervention.

Comparing the two groups with transferrin saturation < 30% and ≥ 30%, no significant differences were observed in age, smoking history, and blood pressure, although among those with transferrin saturation < 30%, more patients were female and had diabetes, a higher BMI, and a history of heart failure. During study initiation, fewer renin–angiotensin–aldosterone inhibitors and less iron were prescribed. No significant differences were observed in eGFR, Hb, or albumin. High-sensitivity CRP and HbA1c levels were significantly higher, and the urinary protein-to-creatinine ratio was significantly lower in the < 30% transferrin saturation group than in the ≥ 30% transferrin saturation group (shown in Table 1).

**Table 1:** Baseline characteristics of the participants by average TSAT < 30% and ≥ 30% during the observation period

	< 30	≥ 30	*P* value
Age, years	69.9 ± 12.0	70.8 ± 11.4	0.243
Male sex, %	50.6	65.7	< 0.001
Diabetes, %	47.3	36.8	< 0.001
Body mass index, kg/m^2^	23.5 ± 4.4	22.7 ± 3.5	0.020
Etiology of chronic kidney disease, %			0.078
Diabetic nephropathy	30.7	24.2	
Nephrosclerosis	25.7	26.4	
Chronic glomerulonephritis	18.9	23.6	
Others	24.7	25.8	
Smoking status, %			0.307
Current	11.1	11.7	
Ever	34.4	39.7	
Cardiovascular disease, %			
Coronary artery disease	17.9	14.3	0.127
Heart failure	9.5	5.2	0.011
Stroke	12.8	11.7	0.636
Peripheral artery disease	9.7	10.3	0.757
Renin–angiotensin–aldosterone inhibitor use, %	63.0	71.1	0.005
Iron supplementation, %	12.8	16.8	0.081
Dose of darbepoetin alfa at 12 weeks (μg)	40.0 (30.0–60.0)	40.0 (30.0–60.0)	0.662
Erythropoietin resistance index	3.92 (2.68–5.93)	3.85 (2.65–5.66)	0.221
Systolic blood pressure, mmHg	134.2 ± 19.2	135.8 ± 19.0	0.151
Diastolic blood pressure, mmHg	71.0 ± 12.3	71.9 ± 12.3	0.141
Creatinine, mg/dL	2.82 ± 1.30	3.03 ± 1.49	0.033
Estimated glomerular filtration rate, mL/min/1.73 m^2^	20.0 ± 9.9	19.2 ± 9.1	0.353
Hemoglobin, g/dL	9.82 ± 0.89	9.78 ± 0.84	0.293
Albumin, g/dL	3.77 ± 0.48	3.70 ± 0.53	0.099
Fe, μg/dL	61.6 ± 19.5	81.3 ± 27.6	< 0.001
Ferritin, ng/mL	106.5 ± 101.8	163.3 ± 160.7	< 0.001
Transferrin saturation, %	22.2 ± 6.9	31.9 ± 10.3	< 0.001
High-sensitivity C-reactive protein, ng/mL	789 (303–2150)	416 (170–1200)	< 0.001
N-terminal-proB-type natriuretic peptide, pg/mL	519 (254–1200)	504 (249–1030)	0.601
Glycated haemoglobin, %	6.19 ± 0.95	5.98 ± 0.88	0.002
Urinary protein–creatinine ratio, g/gCr	2.15 ± 2.78	2.55 ± 3.12	0.005

**Fig. 1:**
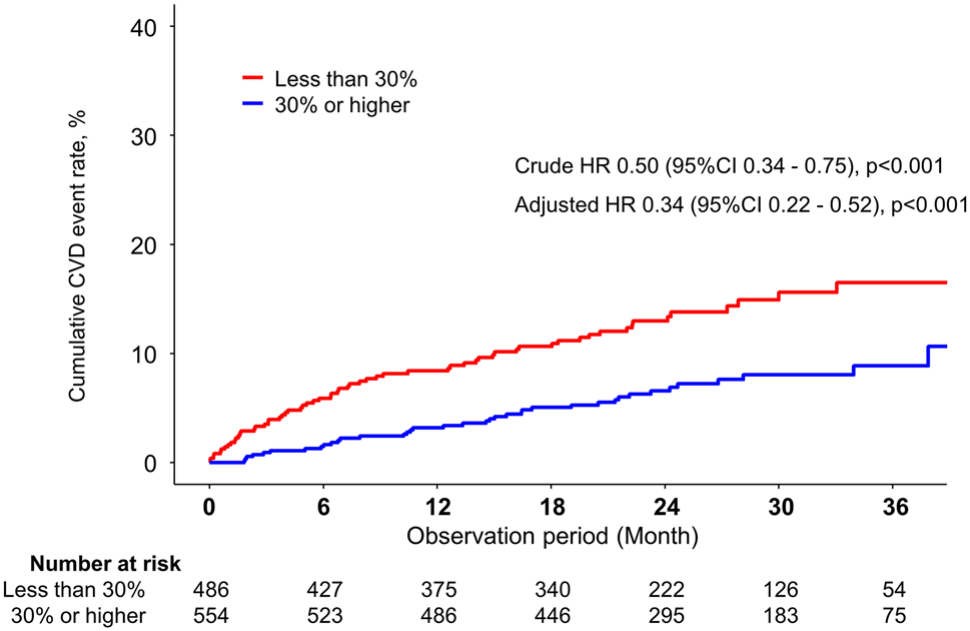
Kaplan–Meier curves for cardiovascular disease incidence classified by transferrin saturation (TSAT) levels > 30% and < 30%. The hazard ratio was adjusted for the following covariates: age, sex, diabetes, body mass index, etiology of chronic kidney disease, smoking status, coronary artery disease, heart failure, stroke, peripheral artery disease, renin–angiotensin–aldosterone inhibitors, iron supplementation, laboratory data at baseline [estimated glomerular filtration rate (eGFR), hemoglobin, albumin, high-sensitivity C-reactive protein, N-terminal-proB-type natriuretic peptide (NT-proBNP), glycated hemoglobin (HbA1c), urinary protein-to-creatinine ratio]

As shown in Fig. 1, the survival curve for cardiovascular events indicated that patients with transferrin saturation ≥ 30% had a significantly better prognosis crude HR of 0.50 (95%CI: 0.34–0.75, *P* < 0.001), which was significant even with an adjusted HR of 0.34 (95%CI: 0.22–0.52, *P* < 0.001). In the stratified analysis, an interaction was identified in the presence of diabetes and iron supplementation, while no interaction was shown in ferritin, Hb, and high-sensitivity CRP (shown in Table 2). As shown in Table 3, when stratified by transferrin saturation levels (< 20%, 20–30%, 30–40%, and ≥ 40), the risk of cardiovascular events was significantly lower in transferrin saturation of 30–40% and ≥ 40% than that of 20–30% after adjusting for multivariate covariates. Furthermore, survival curve analysis for heart failure events revealed that patients with transferrin saturation levels ≥ 30% had a significantly better prognosis than those with transferrin saturation levels < 30%, with a crude HR of 0.42 (95% CI 0.23–0.79, *P* < 0.01), which was significant even with an adjusted HR of 0.26 (95% CI 0.13–0.53, *P* < 0.001) (shown in Supplemental Fig. 4).

**Table 2:** Multivariable-adjusted hazard ratios for cardiovascular events according to transferrin saturation > 30% or < 30% in the subgroups stratified according to baseline characteristics and iron supplementation

		*n/N*	Adjusted hazard ratios (≥ 30/ < 30)		*P* for interaction
Ferritin, ng/mL	< 100	50/485	0.38	0.20–0.73	0.896
	≥ 100	38/412	0.35	0.16–0.77	
Hemoglobin, g/dL	< 11.0	62/492	0.28	0.15–0.51	0.853
	≥ 11.0	38/529	0.55	0.26–1.17	
Sex	F	29/430	0.21	0.07–0.63	0.522
	M	74/610	0.37	0.22–0.60	
Diabetes	**N**	**44/606**	**0.15**	0.07–0.32	**0.004**
	**Y**	**59/434**	**0.67**	0.37–1.21	
Iron supplementation	**N**	**77/739**	**0.27**	0.16–0.46	**0.007**
	**Y**	**26/301**	**0.64**	0.25–1.64	
High-sensitivity C-reactive protein, ng/mL	< 600	46/513	0.43	0.22–0.83	0.739
	≥ 600	54/497	0.36	0.19–0.67	
N-terminal-proB-type natriuretic peptide, pg/mL	< 500	19/498	0.61	0.23–1.65	0.065
	≥ 500	81/512	0.35	0.21–0.57	

Statistical significance was defined as *P* = 0.05 (in bold)

**Table 3:** Unadjusted and adjusted hazard ratios of cardiovascular events according to the categorization of transferrin saturation

Transferrin saturation (%)	Hazard ratio	95% confidence interval	*P* value
Unadjusted model			
< 20	0.82	0.41–1.66	0.584
20–30	Ref	–	–
30–40	0.46	0.30–0.73	< 0.001
≥ 40	0.56	0.29–1.06	0.077
Adjusted model			
< 20	1.18	0.56–2.46	0.668
20–30	Ref	–	–
30–40	0.33	0.21–0.54	< 0.001
≥ 40	0.39	0.19–0.77	0.007

## Discussion

The main finding of our study is that a transferrin saturation level < 30% was associated with a significantly higher incidence of cardiovascular events in patients with CKD receiving an erythropoiesis-stimulating agent. This association was particularly evident in patients without diabetes and in those without iron supplementation. To date, the criteria for iron supplementation have been based on transferrin saturation and ferritin thresholds, with erythropoiesis-stimulating agent reactivity as the primary indicator, and not on cardiovascular events and mortality. Our study found that transferrin saturation levels of 30–40% were associated with a better cardiovascular prognosis independent of ferritin levels in patients with CKD.

Prior to the widespread use of erythropoiesis-stimulating agents, in dialysis patients with markedly elevated ferritin levels, iron deposits were known to cause organ complications such as liver cirrhosis, and ferritin has long been considered an important indicator of iron storage [12, 13]. More recently, in predialysis patients with CKD, ferritin level of at least > 500 ng/mL was associated with a worse prognosis [14]. Although ferritin > 100 ng/mL has reportedly been associated with poor prognosis in patients undergoing dialysis [15], thresholds vary among settings, possibly due to differences in dialysate standards and in prevalence of chronic inflammation [16]. Our study follows the guideline that indicates iron supplementation for CKD patients receiving erythropoiesis-stimulating agents with ferritin levels < 100 ng/mL and withholding iron supplementation for ferritin levels > 300 ng/mL [10]. Furthermore, this recommendation is endorsed by the fact that the erythropoietin resistance index and ferritin had a U-shaped association, with a bottom value of approximately 100 ng/mL in patients receiving long-acting erythropoiesis-stimulating agents [7]. Moreover, patients with transferrin saturation levels < 20% were found to have lower Hb levels, regardless of serum ferritin or CRP levels, while the association between ferritin and Hb was weaker in those with transferrin saturation levels > 20%. Transferrin saturation levels of 30–40% have been associated with the lowest erythropoietin resistance index, regardless of ferritin level. These findings align with the results of our study in pre-dialysis patients. If iron deficiency anemia is defined as the increase in hemoglobin level to the administration of iron, transferrin saturation is a more efficient predictor of “iron deficiency anemia” than ferritin level [17].

Iron deficiency has been associated with ischemic stroke in the general population and with poor prognosis in patients with heart failure [18, 19]. A recent report in patients undergoing dialysis has shown that transferrin saturation of 20–30% was associated with a decreased risk of both cerebrovascular and cardiovascular disease (HR [95% CI] 0.25 [0.07–0.91]), and that transferrin saturation ≥ 30% was associated with a decreased risk of death (HR [95% CI] 0.12 [0.02–0.59]) compared with transferrin saturation that is consistently < 20% [20]. Predialysis CKD patients with the best combination of transferrin saturation and ferritin have the best prognosis, whereas those with lower transferrin saturation and ferritin or higher transferrin saturation and ferritin have a worse prognosis and more hospitalizations for heart failure [14, 21, 22]. In non-dialysis-dependent CKD, absolute and functional iron deficiencies were associated with increased risk of cardiovascular hospitalization, when absolute iron deficiency was defined as transferrin saturation < 20% and/or ferritin < 100 ng/mL and functional iron deficiency as transferrin saturation and ferritin levels of < 20% and 100–500 ng/mL, respectively [14]. In patients undergoing hemodialysis, absolute and functional iron deficiencies were linked to a higher risk of death [23]. However, in this study, absolute iron deficiency was defined as transferrin saturation and ferritin levels ≤ 20% and ≤ 200 ng/mL, respectively, whereas functional iron deficiency was defined as transferrin saturation and ferritin levels ≤ 20% and 200–800 ng/mL, respectively. Transferrin saturation generally maintains a consistent threshold, whereas the ferritin threshold may vary by kidney function, renal replacement therapy, and geographic region. Whether transferrin saturation or ferritin is more prognostically relevant remains unclear. However, the present study found that cardiovascular events decreased with transferrin saturation ≥ 30%, regardless of high or low serum ferritin or CRP levels in patients with non dialysis.

Several mechanisms by which iron deficiency increases the risk of cardiovascular events have been identified. Iron deficiency, as with chronic inflammation, is associated with cardiac hypertrophy and myocardial fibrosis due to the accumulation of increased production and decreased degradation of FGF-23 [24]. According to the previous report, ferric citrate significantly reduces serum phosphate and FGF-23 levels compared to usual care [25]. Unfortunately, our study did not collect data on calcium, phosphorus, PTH, and FGF-23. Of note, a small study found no significant difference in the FGF-23 effect between ferric citrate, a phosphorus adsorbent, and ferrous sulfate, a conventional iron supplement [26]. Ferrous citrate, the iron supplement with the highest number of cases in our study, was analyzed separately and compared with other iron supplements and non-users (shown in Supplemental Fig. 5). The adjusted HR was 0.65 (0.37–1.15, *P* = 0.141) compared to non-users of iron supplements. It has also been reported that iron deficiency through the chelation of iron reduces cardiac contractility through the deterioration of mitochondrial function [27]. Furthermore, iron chelators (deferoxamine) and HIF-PH inhibitors have been reported to induce the calcification of the vascular smooth muscle in an environment with elevated inorganic phosphate [28]. Iron deficiency may pose a risk of cardiovascular events via vascular calcification in patients with CKD, and the importance of iron supplementation is expected to increase as HIF-PH inhibitors become more widely used. Although the exact mechanism is unknown, iron deficiency has been suggested to lead to a hypercoagulable state and increased risk of thrombosis, either through thrombocytosis or by increasing transferrin expression as well as estrogen [29, 30]. These mechanisms suggest that iron deficiency may increase the risk of cardiovascular events. Various studies have reported that iron supplementation corrects hypercoagulability by changing thrombin generation and FXIII activity, inhibiting vascular calcification, and improving renal function and heart failure symptoms [31, 32]. Although it has been reported that intravenous iron supplementation may increase the risk of infection by decreasing the activity of neutrophils, the use of iron-based phosphorus adsorbents, which have recently become widely available, dispels this concern [33, 34].

The limitation of this study is that it was an observational study of guideline-based anemia management with darbepoetin alfa rather than a randomized controlled trial (RCT) with separate groups for transferrin saturation management goals. Although only a few studies exist on transferrin saturation and prognosis in patients with non-dialysis CKD, reports of transferrin saturation of < 10% being associated with a poor prognosis in patients with early-stage CKD without anemia have shown no anemia-related therapeutic intervention; in a large observational study of transferrin saturation < 15% being associated with shorter life expectancy and major adverse cardiovascular events, the proportion of anemia requiring therapeutic intervention was low, 13% with erythropoiesis-stimulating agents and 21% with iron supplements. Therefore, this study is considered a vital report in real-life clinical practice showing the association between transferrin saturation and patient prognosis in treating anemia with erythropoiesis-stimulating agents in patients with CKD. Although future studies, including RCTs, are needed to determine whether higher transferrin saturation can improve prognosis, this study indicates that a higher threshold of 30% transferrin saturation may change the prognosis.

In conclusion, cardiovascular events were significantly lower in CKD patients treated with erythropoiesis-stimulating agents with transferrin saturation ≥ 30% than in those with transferrin saturation < 30%. After multivariate adjustment for potential confounders, cardiovascular events were still significantly lower in patients with a transferrin saturation of 30–40% than in those with a transferrin saturation of 20–30%, suggesting that maintaining transferrin saturation higher than the current practice may be beneficial.

## Supplementary Information

Below is the link to the electronic supplementary material.

Supplemental Fig 1. Flow of participants (TIF 719 KB)

Supplemental Fig 2. Drug prescription and its course in iron supplementation (TIF 590 KB)

Supplemental Fig 3. The course of serum levels of ferritin (a) and transferrin saturation (TSAT) (b) with and without iron supplementation for approximately 12 weeks (TIF 701 KB)

Supplemental Fig 4. Kaplan–Meier curves for heart failure incidence classified by transferrin saturation (TSAT) levels >30% and <30%. The hazard ratio was adjusted for the same covariates as shown in Figure 1 (TIF 589 KB)

Supplemental Fig 5. Kaplan–Meier curves for cardiovascular disease incidence in the following three groups: ferrous citrate, other iron agents, and non-user groups. The hazard ratio was adjusted for the same covariates as shown in Figure 1 (TIF 696 KB)

## Data Availability

The data underlying the results presented in this study are available from the data center for this study, the Translational Research Center for Medical Innovation, Kobe, Japan (https://www.tri-kobe.org/), or from the supporting information files in the following study (10.1371/journal.pone.0277921#sec013) (5).
