# Supplementary figures and images for: Impact of transferrin saturation on cardiovascular events in non-dialysis-dependent chronic kidney disease patients treated with darbepoetin alfa
Source: J Nephrol. 2024 Jun 28;37(8):2327–35. doi: 10.1007/s40620-024-02000-y (PMC11649776; doi:10.1007/s40620-024-02000-y)

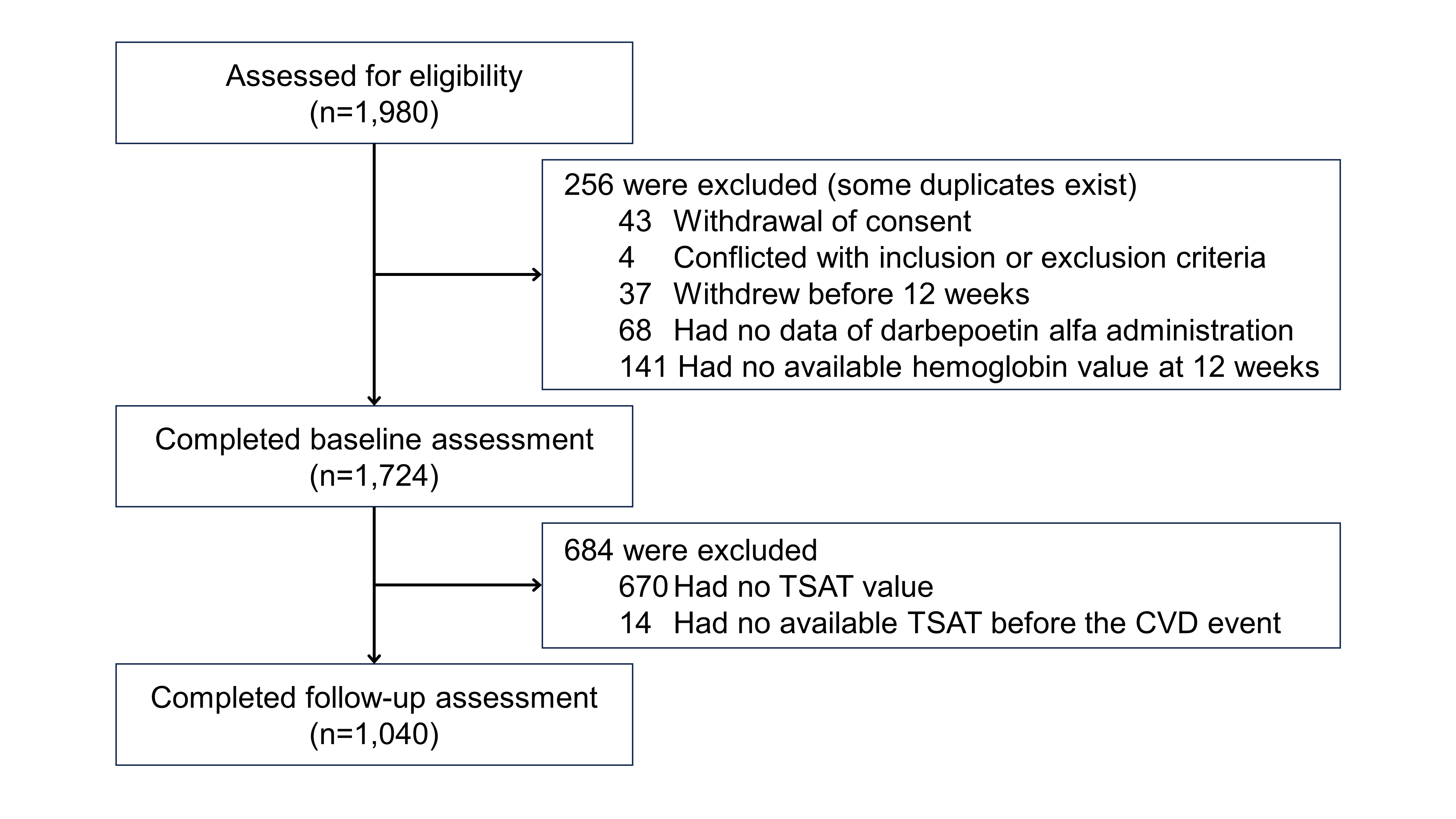

Supplement: Supplementary file 1 — Supplemental Fig 1. Flow of participants (TIF 719 KB) [file 40620_2024_2000_MOESM1_ESM.tif]

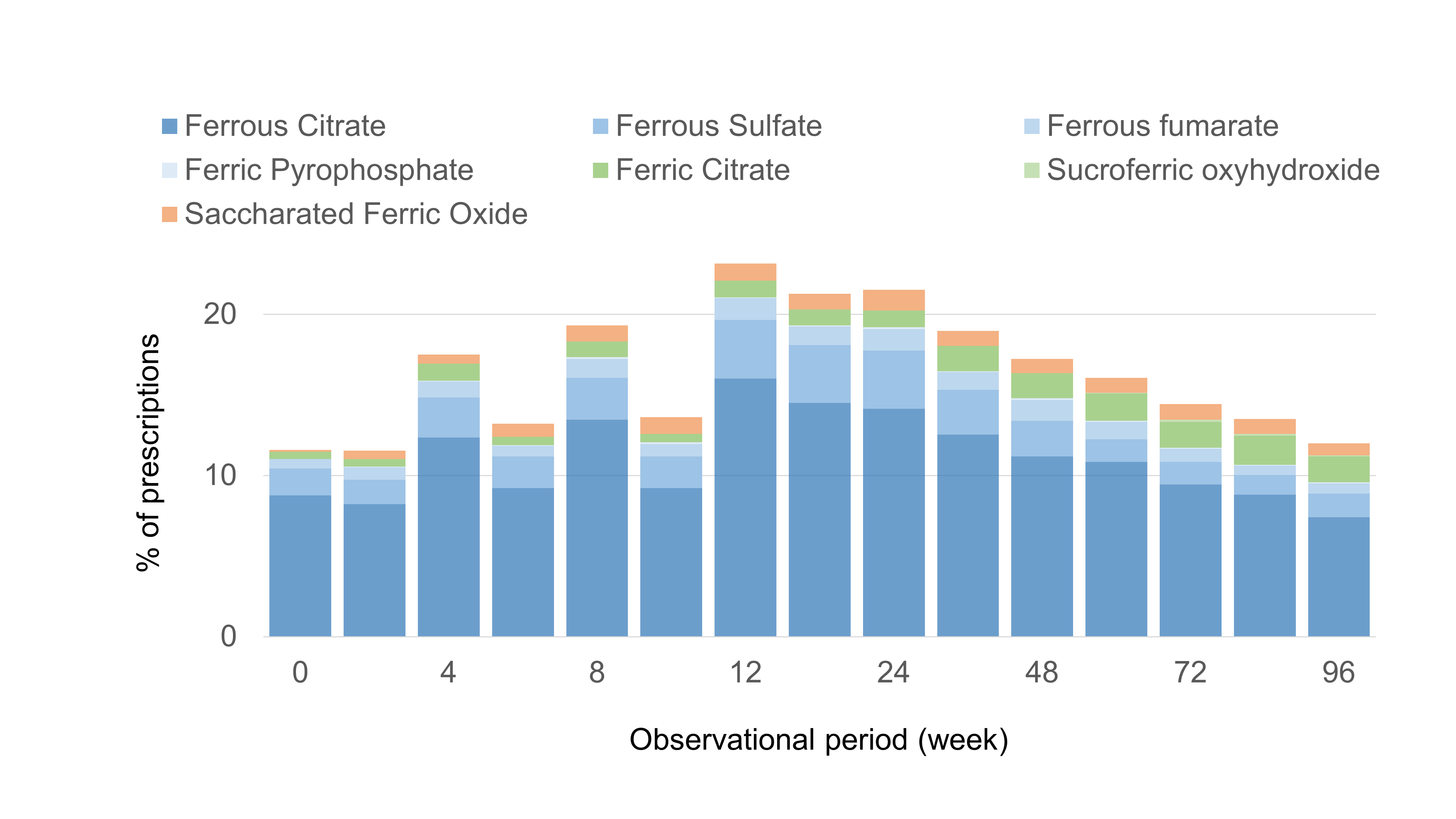

Supplement: Supplementary file 2 — Supplemental Fig 2. Drug prescription and its course in iron supplementation (TIF 590 KB) [file 40620_2024_2000_MOESM2_ESM.tif]

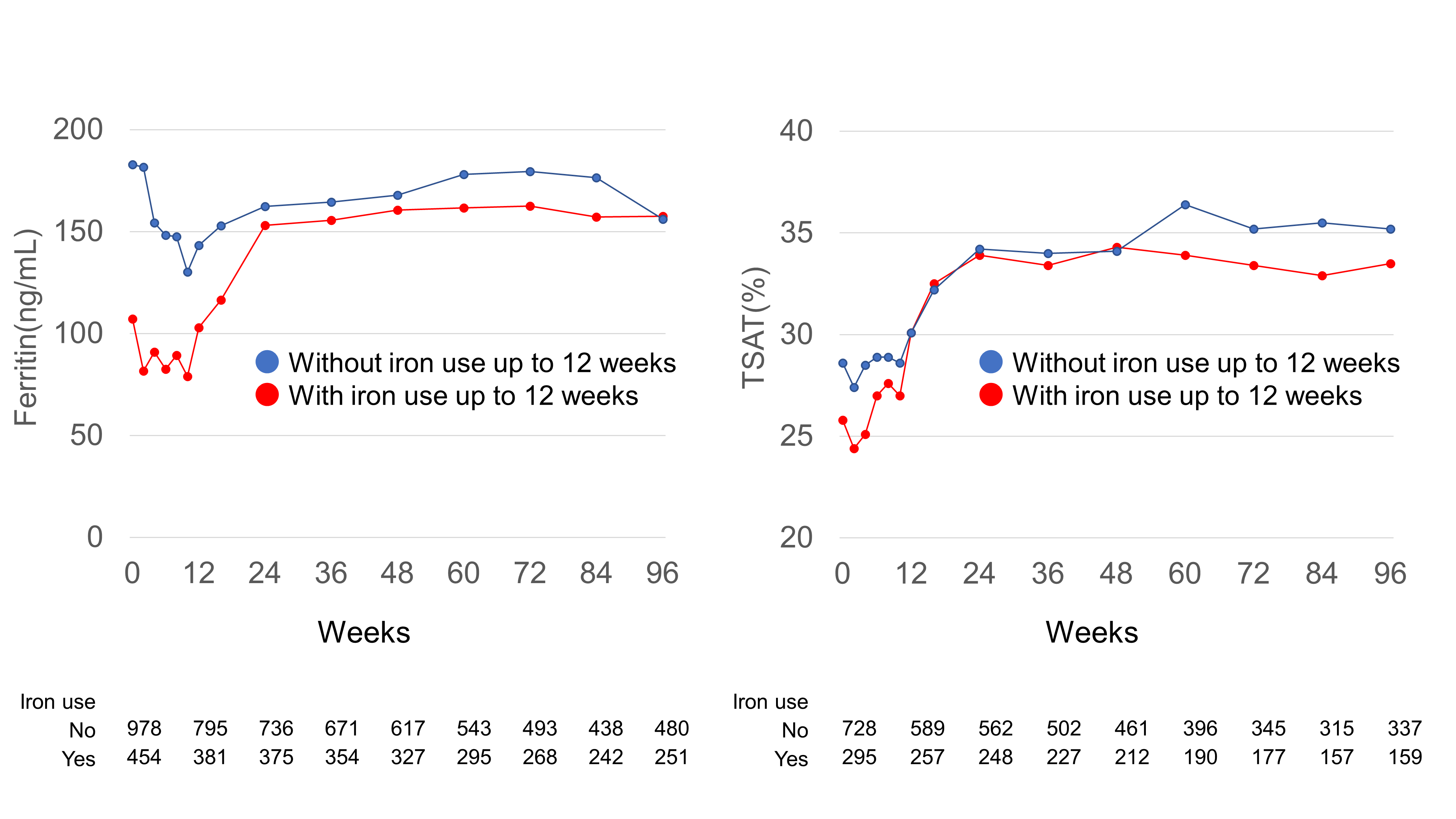

Supplement: Supplementary file 3 — Supplemental Fig 3. The course of serum levels of ferritin (a) and transferrin saturation (TSAT) (b) with and without iron supplementation for approximately 12 weeks (TIF 701 KB) [file 40620_2024_2000_MOESM3_ESM.tif]

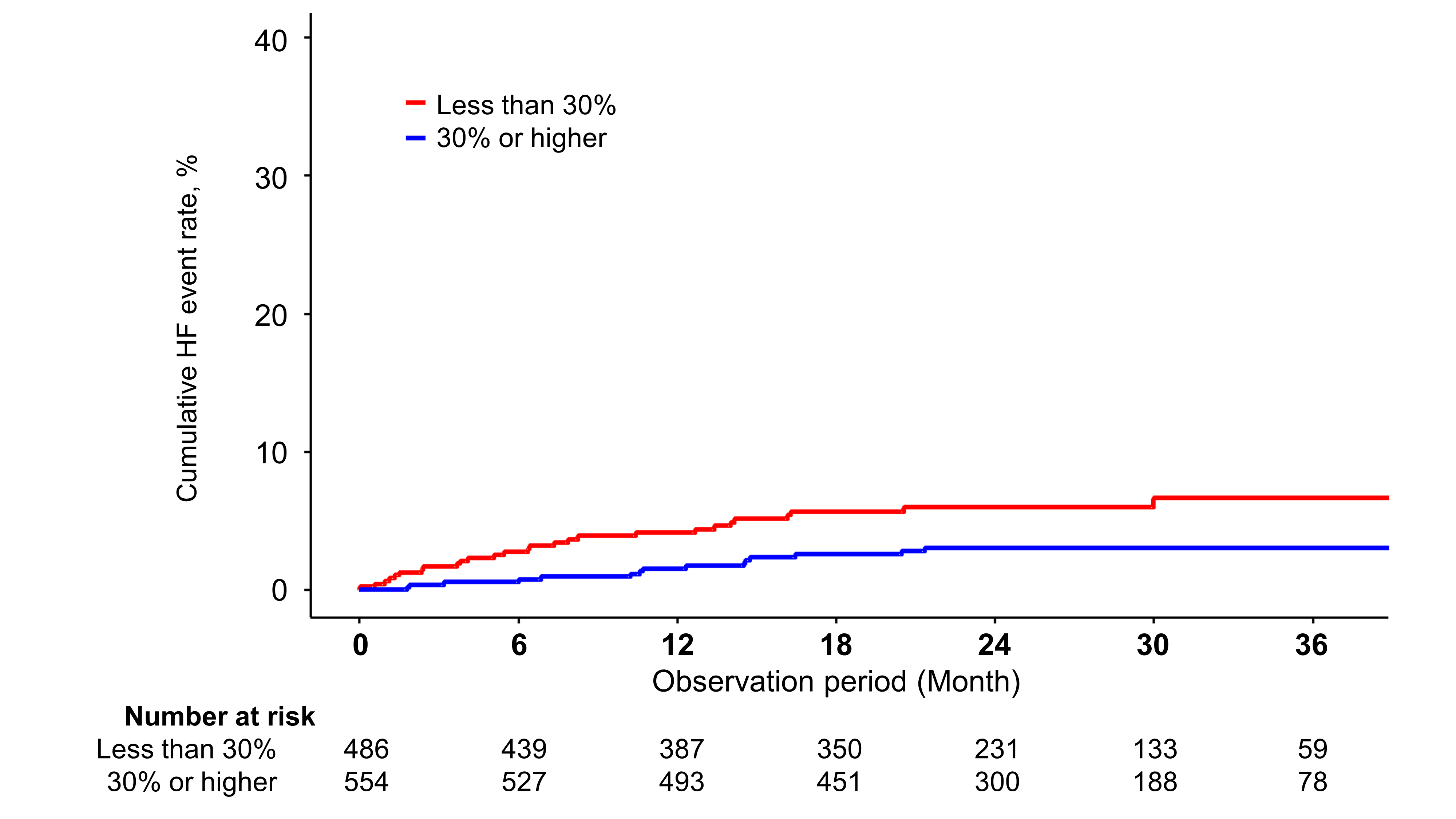

Supplement: Supplementary file 4 — Supplemental Fig 4. Kaplan–Meier curves for heart failure incidence classified by transferrin saturation (TSAT) levels >30% and <30%. The hazard ratio was adjusted for the same covariates as shown in Figure 1 (TIF 589 KB) [file 40620_2024_2000_MOESM4_ESM.tif]

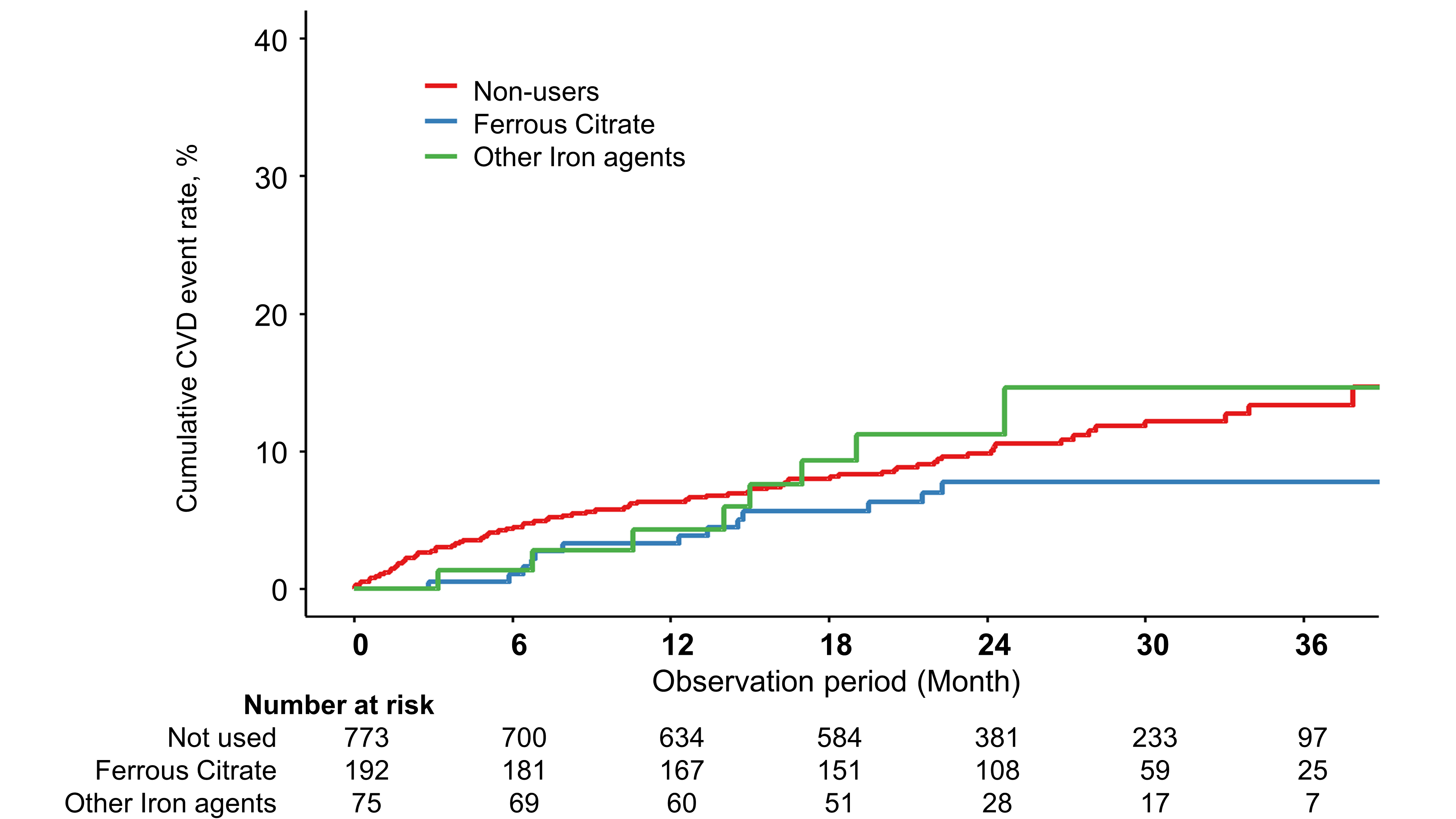

Supplement: Supplementary file 5 — Supplemental Fig 5. Kaplan–Meier curves for cardiovascular disease incidence in the following three groups: ferrous citrate, other iron agents, and non-user groups. The hazard ratio was adjusted for the same covariates as shown in Figure 1 (TIF 696 KB) [file 40620_2024_2000_MOESM5_ESM.tif]
